# A Theory-Based Contextual Nutrition Education Manual Enhanced Nutrition Teaching Skill

**DOI:** 10.3389/fpubh.2018.00157

**Published:** 2018-05-29

**Authors:** Mojisola D. Kupolati, Una E. MacIntyre, Gerda J. Gericke

**Affiliations:** Human Nutrition, Faculty of Health Sciences, University of Pretoria, Pretoria, South Africa

**Keywords:** nutrition education manual, teachers, learners, social cognitive theory, meaningful learning model

## Abstract

**Background:** A theory-based contextual nutrition education manual (NEM) may enhance effective teaching of nutrition in schools. School nutrition education should lead to the realization of such benefits as improved health, scholarly achievement leading to manpower development and consequently the nation's development. The purpose of the study was to develop a contextual NEM for teachers of Grade 5 and 6 learners in the Bronkhorstspruit district, South Africa, and to assess teachers' perception on the use of the manual for teaching nutrition.

**Methods:** This descriptive case study used an interpretivist paradigm. The study involved teachers (*N* = 6) who taught nutrition in Life Skills (LS) and Natural Science and Technology (NST) in a randomly selected primary school in the Bronkhorstspruit district. Findings from a nutrition education needs assessment were integrated with the constructs of the Social cognitive theory (SCT) and the Meaningful learning model (MLM) and the existing curriculum of the Department of Basic Education (DoBE) to develop a contextual NEM. The manual was used by the teachers to teach nutrition to Grades 5 and 6 learners during the 2015 academic year as a pilot project. A focus group discussion (FDG) was conducted with teachers to gauge their perceptions of the usefulness of the NEM. Data were analyzed using the thematic approach of the framework method for qualitative research.

**Results:** Teachers described the NEM as rich in information, easy to use and perceived the supporting materials and activities as being effective. The goal setting activities contained in the NEM were deemed to be ineffective. Teachers felt that they did not have enough time to teach all the important things that the learners needed to know.

**Conclusion:** Teachers perceived the NEM as helpful toward improving their nutrition teaching skills.The NEM template may furthermore guide teachers in planning theory-based nutrition lessons.

## Introduction

A nutrition education (NE) manual is a step by step guideline and plan for delivering nutrition lessons using theoretical concepts (1). The step by step guidelines for delivering nutrition messages can also be referred to as a curriculum, a lesson plan or an educational plan (2). Theory based, behaviorally oriented methods are known to be effective in NE (3). In the debate on the usefulness and barriers of theory application in nutrition interventions, Brug and colleagues argued that theories promoting behavior change and motivation are useful (4). Theories may bridge the gap between motivation for change and behavior change (5). Bridging the gap between motivation and behavior change is especially important in nutrition interventions. Nutrition education curriculum development and implementation based on behavioral theories such as social cognitive theory (SCT) and the meaningful learning model (MLM) has been successful (6, 7). Successful school NE curricula, incorporating SCT, capitalized on the influence of the school environment on learners' ability to learn new behaviors (6–9). The MLM encourages active involvement of learners in the delivery of messages by ascertaining learners' prior knowledge, establishing and facilitating the use of new learning in real life situations (7, 10).

Nutrition education curricula are usually developed to address specific issues. For example, the Food and Agriculture Organization's (FAO) NE curriculum took into account that many developing countries do not offer NE in schools (1). In both developing and developed countries, NE curricula were developed to improve school children's attendance and attention in class, to develop healthy eating, and to promote healthy food behaviors (1, 11, 12).

Nutrition teaching is generally included as topics within subjects, focusing on increasing learners' knowledge of good nutrition, often neglecting behavior change (1, 13, 14). In South Africa, the Department of Basic Education (DoBE) emphasized behavioral outcomes in the curriculum but the did not include a behavioral component (15). Nutrition curricula without behavioral components may undermine the benefits of school NE (6, 16–18). The literature on school NE in South Africa has documented several important issues. These concerns included a need for an educational strategy that fits into the school curriculum, unhealthy eating among learners, and a need for an effective approach for communicating nutrition messages to learners from limited resource settings (19, 20). Additionally, there is limited information in the literature regarding the details of the educational materials used in curriculum-based NE interventions; the integration of behavioral and learning theories is often not explained. Against this background, we developed a theory based contextual nutrition education manual (NEM) to promote healthy eating. We assessed if teachers who used the NEM deemed the resource as valuable. The manual was developed by enhancing the existing DoBE curriculum using SCT and MLM. To our knowledge, this is the first project to execute school NE with this approach in Africa.

## Methods

### Needs assessment

Using qualitative and quantitative methods, we assessed the NE needs of primary school teachers and learners in the Bronkhorstspruit district (18, 21, 22). The nutrition knowledge, attitudes and dietary practices (KAP) of teachers and learners, as well as the NE practices of the teachers, were assessed. We explored the perceptions of teachers regarding the influence of school NE on the eating behaviors of learners in focus group discussions (FDGs).

The needs assessment revealed two categories of factors that may influence behavior:

Factors with the potential of promoting healthy eating:

- locally available foods in the community,
- foods served through the National School Nutrition Program (NSNP),
- healthy choices of foods in tuck shop,
- teachers' desire for training to teach nutrition,
- classroom nutrition messages.

Factors with the potential of hindering healthy eating:

- lack of teachers' nutrition knowledge in certain areas such as the energy content of food, sources of nutrients and composition of fats,
- unavailability of up to date instructional materials such as teachers' manuals, posters, learner's workbooks and hands-on materials,
- lack of nutrition training for the teachers,
- inappropriate methods of delivery of NE to learners,
- learners' inadequate nutrition knowledge,
- learners' resource-constrained environment,
- unhealthy choices of foods among the learners.

### Development of the manual

The DoBE curriculum for Grades 5 and 6 outline nutrition topics in two subject areas. The topics were “Healthy eating for children” in Life Skills (LS) in Grade 5, “Food hygiene” in LS in Grade 6, and “Nutrients in food,” “Nutrition,” and “Food processing” in Natural Science and Technology (NST) in Grade 6 (15, 23). We modified these topics by using selected constructs of the SCT and the MLM to explain the nutrition concepts. For example, we identified factors facilitating healthy eating such as foods that were locally available and used these as examples in class lessons, practical classes, and demonstrations. Furthermore, the existing curriculum was enhanced with the desired behavioral change components to address the challenge of inappropriate methods of delivery of NE. A theory based template was developed for each nutrition topic as described in an example of the template for Grade 5 (Table 1).

**Table 1:** Example of template for grade 5 nutrition topic—Healthy Eating for Children.

**Contextual meaning of theory construct**	**Application of constructs**	**Expected learner outcomes**
**Environment (SCT)**		
External factors which may influence learners' eating behaviors	-Used available foods in the community as examples in explaining the SAFBDG, and provided effective ways to use the foods	-To identify and use foods that are good choices in the community
**Behavioral capability (SCT)**		
Learners receive nutrition messages and acquire healthy eating behaviors	-Delivered messages of the SAFBDG using an active participatory approach	-To choose and enjoy different foods in their meals
	-Provided healthy alternatives to using foods that were nutrient poor, but high in fat, sugar or salt	
**Expectation (SCT)**		
Learners anticipate outcomes of healthy eating	-Learners identified and drew their aspirations (What they will like to become)	-To anticipate achievement of their aspiration by enjoying different foods
**Observational learning (SCT)**		
Learners acquire behavior by watching the actions of role models (teachers, peers and parents) who perform the behaviors	-Teachers displayed foods like oranges, apples, carrots and brown bread on his/her desk always	-To learn to enjoy different foods by watching teachers -To learn from the successes of peers
	-Learners shared experiences of healthy eating	
**Self-regulated learning (SCT)**		
Create an opportunity for self-monitoring of learners through goal setting	-Learners set goals to implement the guidelines for healthy eating	-To identify barriers to implementing the guidelines for healthy eating
		-To identify and use healthy alternatives
**Relevant prior knowledge (MLM)**		
Establish learners' prior knowledge for meaningful learning to occur	-Revised previous lessons on healthy and unhealthy eating	-To consolidate previous knowledge about healthy and unhealthy eating
**Integrate new with prior knowledge (MLM)**		
Teachers and learners build on existing knowledge of healthy eating behaviors	-Integrated healthy and unhealthy eating in the messages of SAFBDG	-To apply the knowledge about healthy and unhealthy eating and the messages of SAFBDG to make healthy food choices

*SCT, Social cognitive theory; MLM, Meaningful learning model; SAFBDG, South African Food-Based Dietary Guidelines*.

The manual included a picture book, learner's workbook, and posters. The NE materials were subjected to face and content validity by the officers of the DoBE and nutrition experts at the University of Pretoria. The DoBE officers recommended limiting the manual to essential concepts suited to the allotted 30 min lesson time. We shortened the manual to comply with the suggestions made by the DoBE officers.

### Nutrition education manual and the accompanying materials

The NEM featured the nutrition topics for Grades 5 and 6, outlined in the DoBE's curriculum and delineated into sub-topics to make up a chapter. Each chapter consisted of a sub-topic plan with an outline and subtopic notes. The sub-topic plan provided teachers with guidelines on how to present nutrition messages to learners while accommodating the learners' resource-constrained situation. The sub-topic notes provided detailed information about the sub-topics and served as a resource when the teachers prepared for teaching the lessons.

We presented the sub-topic plan and outline in clear headings which described the pedagogy for implementing the lessons: objectives, lesson preparation, learners' prior knowledge, active participatory teaching and learning, problem-based small group discussion and self-regulatory learning. The self-regulatory learning consisted of two activities for learners. Firstly, the learners' goal setting exercises where learners made decisions concerning the nutrition messages they received and secondly the learner's worksheets where learners engaged in problem identification and problem-solving exercises. The instruction approach featured educational activities such as class demonstrations with food items, correct hand washing techniques, and learners' goal setting. Learners were encouraged to involve their parents by sharing their worksheet exercises and goals with families. The pedagogical outline of the NEM in one of the nutrition lessons is presented in Table 2.

**Table 2:** Pedagogical outline of the NE manual in a lesson “South African Food-Based Dietary Guidelines.”

**Pedagogy specifics**	**Strategies**	**Educational activities**
**Preparation**		
Lesson objectives (e.g., To enable learners to understand and apply the guideline for enjoying a variety of foods) Lesson notes Posters	Teacher's preparation for the lesson	Displaying healthy choices of food on her desk and posters on the classroom walls Updating personal nutrition knowledge
**Learners' prior knowledge**		
Refreshing learners' knowledge of dietary habits of children	Teachers revise previous lessons and confirm what learners already know	Revising the previous lesson using the question-answer method
**Active participatory teaching and learning**		
Presenting the healthy eating messages of the SAFBDG using question-answer method	Teachers employ active participatory teaching and learning	Incorporating foods commonly eaten in the community as examples
**Problem-based small group discussion**		
Learners' group work: Applying the principles of the SAFBDG Creating mixed meals from different foods Listing the food groups in the SAFBDG	Learners engage in problem-based small group discussion	Building learners' skills by engaging them in problem identification (e.g barriers to having healthy snacks) and solution (e.g., replace corn chips with a fruit)
**Goal setting**		
Lessons learned Decisions made	Learners set goals	Encouraging learners to complete the goal setting section with parent/guardian assistance
**Worksheet—Aspiration**		
(What learners aspire to become in future) Drawing the aspiration Fuelling the aspiration	Learners perform lesson activities on the worksheets	Including class/home works that help learners to apply their prior and new knowledge in real life situation

*SAFBDG, South African Food-Based Dietary Guidelines*.

The picture book was a collection of pictures relevant to the learners' context to be used as a supplementary resource.

The learner's workbook was a collation of the learners' tasks and comprised goal setting exercises and worksheet activities that learners were to complete with parents or guardians at home. We structured the NEM in such a way that teachers had to encourage learners to set their goals. Teachers had to monitor learners' progress toward achieving their goals.

The posters were pictorial illustrations of nutrition concepts. Teachers could hang posters on classroom walls serving as constant reminders of nutrition messages.

### Participants

The Gauteng DoBE selected three primary schools to participate in a broader study: “Schools as sites for social change” also an Institutional Research Theme (IRT) of the University of Pretoria. One of the three primary schools was randomly selected to pilot the developed NEM. The teachers (*n* = 6) for LS and NST in Grades 5 and 6 in the selected school were purposively selected to participate in the study. Four teachers taught nutrition through LS and NST subjects to 177 6th Grade learners in five classes, and two teachers taught nutrition through LS to 173 5th Grade learners in five classes. All of the teachers consented to participate in the study.

### Intervention

We trained the teachers to use the NEM in a 1 day workshop, which gave opportunities for familiarization and obtaining input from the teachers on the outline and practical implementation of the manual. During the workshop, we taught fundamental nutrition concepts to the teachers, addressing one of the needs identified in the needs assessment. After the workshop, teachers used the manual to teach nutrition topics in the DoBE curriculum for one academic year (2015). Each learner received a workbook to complete class and homework activities.

At the end of the academic year, a focus group discussion (FDG) with the teachers was conducted to obtain their perceptions of the use of the NE manual. The small sample of teachers necessitated a single FGD. A researcher and two research assistants conducted the FGD, which lasted 56 min. The assistants took notes and monitored the audio tape recording according to recommended procedures (24).

### Assessment of perceptions

We developed an interview guide of 12 questions to guide the discussion. Nutrition experts at the University of Pretoria assessed the face and content validity of the interview guide. Examples of the questions were “In your own opinion, how can the use of the manual in presenting nutrition messages help learners to adopt healthy eating?” and “What part(s) of the lesson did learners enjoy the most?”

### Data analysis

Data were analyzed using the thematic approach of the framework method which comprises data familiarization, thematic pattern identification, theme coding, charting and mapping (25, 26). The emerging themes were discussed in categories.

## Results

The perceptions of teachers regarding the use of the NEM were described using selected quotes designated with the teachers' participating number (e.g., P2—participant 2). The teachers' views of the use of the NEM were discussed under three themes, namely the perceptions of the manual, the perceptions of the supporting materials and activities and recommendations for improvement.

### Teachers' perceptions of the manual

The teachers described the manual as rich in information, well-outlined with nutrition concepts, adequately illustrated with colorful pictures and as easy to use. They explained that the cover page pictures illustrated healthy eating and were relevant to the learners' environment. The teachers remarked:

“*It was easy to use the manual and to understand. The mere looking at the manual with the colorful pictures made it easy to use it to teach.”*—P2.

Teachers viewed that the teaching of nutrition using the NEM might influence learners' eating behaviors in two ways. Firstly, learners would benefit from nutrition knowledge in the future. Teachers explained that learners' low economic background might limit their capacity to effect behavioral change.

“*It would help learners to know what they didn't know before. If they couldn't practice what they learned now because they couldn't afford the foods, they will be able to use the knowledge to benefit their lives in future.”*—P4.

Secondly, teachers felt that learners benefited from the practical demonstrations in which they participated in class. Learners could implement these practices in their homes, for example, the demonstration of effective hand washing.

Teachers felt that the NEM had more information relevant to the learners' situation compared to the DoBE recommended textbooks for nutrition topics.

“*The manual was more practical, the foods mentioned in the manual were what the learners were familiar with which they also used in their homes.”*—P4.

### Teachers' perceptions of the supporting materials and activities

During the FGD, we discussed the usefulness of the posters and picture book in teaching the nutrition lessons. We discussed the influence of goal setting on learners' eating behaviors, the influence of worksheet activities on learners' understanding, the influence of group discussions on learners' understanding and the parts of the lesson that the learners enjoyed the most.

Teachers viewed the posters and the picture book as valuable supplements to the manual because they helped to consolidate the nutrition messages, making teaching lively.

“*The picture book was helpful because when we taught the lessons, we used the pictures in the picture book to show learners examples.”*—P2.

Teachers perceived the goal setting activities as exercises in which the learners had to make decisions beyond their control. Teachers explained that learners only ate what their parents provided, and some learners depended on foods served at school. Teachers, therefore, reasoned that the goal setting activities were unlikely to have an appreciable influence on the learners' eating behaviors.

“*For learners to set their goals was difficult for them because they did not have a choice, it was what their parents gave that they would eat.”*—P5.

Teachers perceived the worksheet activities as being unable to influence learners' understanding. The workbooks contained too few non-writing activities such as crosswords or matching pictures with texts.

“*The worksheets required more of writing which our learners did not like so much. Our learners like to cut and paste pictures and also drawing rather than writing.”—*P4.

The problem-based group discussions presented opportunities for learners to learn from each other while the classroom demonstrations and practical sessions gave opportunities for learners to work in groups.

“*The group discussion helped them to learn because they had the opportunity to share ideas and learned from the ideas of the other members of the group.”*—P2.

Teachers mentioned that learners enjoyed the lessons about the consequences of a poor diet. Teachers reported that learners also enjoyed the practical sessions and demonstrations and that all the learners wanted to participate.

“*The part of demonstrating effective washing of hands was very interesting for learners; they had said we thought when we wet our hands it was clean. They saw that wetting of hands was not clean at all when we did the demonstration.”*—P1.

### Teachers' recommendations for improvement

Teachers recommended improvement in three themes, namely teachers' decision to continue using the manual, the strengths of the manual and suggestions for improvement.

Teachers mentioned that they would like to continue to use the manual to teach nutrition. They felt that the manual had a clear outline, attractive appearance and used simple language. The NEM was thus practical and easy to use. The content was in line with the existing curriculum, and the messages were implementable.

“*If ever it was my choice, I would like that all the other aspects of NST be like this manual.”*—P3.

Teachers emphasized that the strengths of the NEM lay in the implementable messages and attractive presentation with illustrations.

“*It was valuable in the sense that it taught learners what could practically be done.”*—P4.

The teachers suggested the following improvements to the manual: the learner's workbook should include more optional activities and should feature more hands-on activities.

## Discussion

We developed a NEM that capitalized on the strengths of behavioral theories to facilitate improved nutrition knowledge and desirable behavior change. We selected SCT constructs, including the environment, behavioral capability, expectation, observational learning, and self-regulated learning. The MLM constructs included relevant prior knowledge and integration of new knowledge with prior knowledge.

While developing the NEM, we referred to the environment as external factors capable of influencing learners' eating behavior (2). In the needs assessment, we identified the environmental factors with the potential of promoting or hindering healthy eating. We incorporated these factors in the NEM by using foods available in the community as examples, giving alternatives to unhealthy choices of foods and giving ideas on how to increase food availability and variety at home. Previous studies have reported significant improvement in fruit intake in a curriculum-based NE intervention for learners which involved the use of a school garden, lunchroom, school environment and classroom nutrition lessons (27, 28).

Behavioral capability is about acquiring knowledge about an issue and developing the skills to perform the behaviors to address the issue. In this case, if we expect learners to adopt healthy eating behaviors, they need to know what healthy eating is and how to eat healthily (29). We integrated behavioral capability into the curriculum through interactive and question-answer methods of lesson delivery. We recommended that learners actively engage in classroom activities and interpret class experiences for making healthy food choices (1).

In the NEM, expectation concept was presented by emphasizing positive outcomes of healthful behaviors. Learners who can identify positive outcomes are more likely to adopt healthy eating behaviors in both the short and the long term. Knowing that healthy eating promotes brain function and good school performance is a powerful motivator for learners (30). Other motivators included in the NEM were good growth, healthy bones, healthy teeth and to be disease free.

Observational learning, or acquiring behavior through watching the actions and outcomes of others performing the behavior (2), was facilitated in the manual by including credible role models (the teachers, parents, and peers) of healthy eating. In the NEM, we recommended that teachers and parents be encouraged to model healthy eating for learners (1, 31).

Self-regulated learning was integrated through goal setting activities included in the learner's workbook. The workbook encouraged learners to attain their goals by committing to a line of action (29). The goal setting activities required learners to evaluate their knowledge, then write their goals and plan strategies on how to accomplish their eating goals.

In principle, the MLM states that learning takes place primarily by building on existing knowledge which eventually brings about new understandings (32). We incorporated the relevant prior knowledge concept of MLM into the NEM by checking learners' understanding of new lessons and by revising previous lessons with learners (33). The concept of integrating new with prior knowledge advocates building on learners' existing nutrition knowledge (7). The workbook integrated new with prior knowledge via workbook activities and class demonstrations, where learners added new knowledge to what they already knew and applied the knowledge in real life situations by solving food-related problems.

The developed NEM featured curriculum structures such as objectives, group discussions, prior relevant knowledge and building on existing knowledge as reported in previous studies (7, 33).

Developing NE curricula using SCT and the MLM may facilitate more effective learning. Research efforts involving these theories provided useful insights. An intervention encouraging the intake of fruits and vegetables among grade five and six learners used a 10-week curriculum intervention based on SCT (34). The learners ate significantly more fruits and vegetables after the intervention (34). Babadogan and colleague used the concept of meaningful learning to present examples of instructional design for Social Studies compared to information processing theories. Meaningful learning was expository favoring students' active participation in class (7).

Our NEM enhanced the nutrition topics outlined by the DoBE following the allotted time. This strategy corroborates the findings from previous studies that integrating school-based NE into the existing curriculum brings about desirable results (6, 35, 36). Also, supporting the developed NE manual with materials, i.e., the picture book, learner's workbook and the posters is consistent with the recommendations that school NE materials should comprise a teacher's manual and instructional materials (35).

The teachers described the manual as informative, well-illustrated with pictures and easy to use. In previous studies, teachers valued clear instructions and good arrangement as helpful teaching aids (37). The components of a NEM and ease of use are some of the factors that could motivate teachers in implementing NE programs (38, 39). In this study, teachers perceived that the NEM could influence learners eating behavior in two ways. Firstly, learners had learned valuable nutrition concepts and would be able to use this knowledge in the future. Teachers felt that learners' current resource-constrained situation limited their ability to make immediate behavioral changes. A constrained socio-economic background may hinder the success of school NE interventions (18, 40, 41). In resource-limited communities, NE curricula need to be carefully considered to have the desired outcomes. For example, if the supply of healthy food is limited, learners need to be taught how to grow seasonal crops or develop home gardens to improve food variety and household food security. McNulty reasoned that school NE should prepare learners for their role as future parents who need to take responsibility for their own nutritional well-being and that of their families (14). It may be frustrating for learners to learn about nutrition if they are unable to use the information in their resource-constrained situations. Furthermore, learners who drop out of school early to start a family may benefit from nutrition knowledge acquired during school NE (42).

Secondly, learners benefited from some of the classroom demonstrations such as effective hand washing. This lesson could also be applied at home.

In this study, teachers felt that the NEM was more valuable than previously used textbooks. The NEM was contextual in nature and rich in information with a structured instructive outline. Teachers also valued the supporting materials of the NEM, i.e., the picture book, posters, and the workbook, and the activities such as the learners' goal setting and group discussions. In a previous study, a detailed NEM and lesson plan encouraged teachers to facilitate a school NE program (39). In this study, teachers had mixed feelings about the learners' goal setting, group discussions and workbook activities translating into healthy eating in the present situation. The teachers perceived that learners enjoyed the practical demonstrations, the content on the consequences of poor diets and the drawing of their future aspirations. Previous studies have reported that learners always show more interest when learning is accompanied by activities rather than through instruction only (40, 43).

Teachers indicated their willingness to continue using the NEM if given the opportunity. The reasons given emphasized the fact that teachers appreciated the manual's attractive appearance, simple language, practical orientation, and ease of use. In previous studies, teachers gave similar reasons when expressing their satisfaction with the educator's manual and the NE materials (39, 41).

The strengths of the NEM such as its integration in the existing curriculum and its well-structured pedagogy for use by teachers are valuable and contribute to the potential sustainability of the NEM. Teachers suggested that the learner's workbook should include more activities from which teachers could choose and that the learner's workbook should include more hands-on activities because learners tend to sustain knowledge after engaging in practical activities. Teachers recommended that supporting materials for school NE should include a variety of resources in different presentations to enhance effective teaching of nutrition in schools (35). Teachers suggested that posters could be improved through lamination, further revealing the teachers' desire to continue using the NEM.

This evaluation was limited to a single FGD with six teachers. Interviewing a larger number of teachers would have allowed for more FGDs and provided richer information. The findings of this study cannot be generalized to entire South Africa due to the small sample size and the contextual orientation of the NEM. However, as a pilot project, this study has provided direction to scaling up activities. Moreover, the NEM was based on the national curriculum which is used by all public primary schools in South Africa.

## Conclusion

This study demonstrated the feasibility of developing a theory-based contextual NE manual based on an existing school curriculum. Teachers perceived the contextual NEM to be helpful for teaching nutrition to learners. While developing the NEM, we considered and used selected constructs of the SCT and the MLM to enhance the existing curriculum for use in resource-limited settings. We demonstrated that these theories improved the understanding of the concepts of nutrition in the context of the teachers' experiences.

Future studies could continue to implement the NEM to more schools, including all Grades. Teachers could use the nutrition lesson template to guide the planning of nutrition lessons suited to learners' specific needs. The theoretical template could be used to adapt the NEM to different settings with special attention to the prevailing situation of the target audience.

## Ethics statement

This study was carried out in accordance with the recommendations of the Gauteng Department of Education (Numbers: D2014/199; D2014/308A and D2015/374A). The protocol was approved by the Research Ethics Committee of the Faculty of Natural and Agricultural Sciences of the University of Pretoria (Number: EC130424-037). All participants provided written informed consent in accordance with the Declaration of Helsinki.

## Author contributions

UM and GG conceptualized the study. MK collected the data and wrote the first draft of the manuscript with contributions from UM and GG. All authors reviewed and approved the draft of the manuscript and ensured the accuracy and integrity of the work before submission to the journal.

### Conflict of interest statement

The authors declare that the research was conducted in the absence of any commercial or financial relationships that could be construed as a potential conflict of interest.
